# A 5-year evaluation of quality of life, pelvic discomfort, and sexual function following posterior pericervical repair

**DOI:** 10.25122/jml-2023-0321

**Published:** 2024-04

**Authors:** Zinat Ghanbari, Parivash Jelodarian, Fatemeh Hosseini Salkisari, Samira Sohbati, Tahereh Eftekhar, Reihane Sadat Hosseini, Zahra Nezami, Maryam Deldar Pesikhani

**Affiliations:** 1 Department of Obstetrics and Gynecology, Pelvic Floor Fellowship, Faculty of Medicine, Tehran University of Medical Sciences, Tehran, Iran; 2 Pelvic Floor Fellowship, Department of Obstetrics and Gynecology, Fertility Infertility and Perinatology Research Center, School of Medicine, Ahvaz Jundishapur University of Medical Sciences, Ahvaz, Iran; 3 Pelvic Floor Fellowship, Department of Obstetrics and Gynecology, Faculty of Medicine, Tehran University of Medical Sciences, Tehran, Iran; 4 Department of Obstetrics and Gynecology, Clinical Research Development Unit, Afzalipour Hospital, Kerman University of Medical Sciences, Kerman, Iran; 5 Fellowship of Female Pelvic Medicine and Reconstructive Surgery, Department of Obstetrics and Gynecology, School of Medicine, Shahid Sadoughi University of Medical Sciences, Yazd, Iran

**Keywords:** Lower Urinary Tract Symptoms Module (ICIQ-FLUTSsex), Pelvic Floor Distress Inventory-20 (PFDI-20), pelvic organ prolapse, prolapse surgery

## Abstract

The aim of this study was to evaluate the quality of life, pelvic discomfort, and sexual function of patients who underwent posterior pericervical repair or level I to III surgical procedures for pelvic organ prolapse (POP) after 5 years of follow-up. This retrospective cohort study enrolled 107 women with POP who were referred to the Imam Khomeini Hospital Complex, an academic center affiliated with the Tehran University of Medical Sciences, Tehran, Iran, from 2014 to 2021. The patients underwent transvaginal surgery using native tissue, in which the rectovaginal fascia was attached to the pericervical ring. The Pelvic Floor Distress Inventory-20 (PFDI-20) and Lower Urinary Tract Symptoms Module (ICIQ-FLUTSsex) questionnaires were completed by each patient before and 5 years after surgery. Of the 107 patients, only 78 completed the 5-year follow-up. The mean PFDI-20 scores before, 12 months, and 5 years after surgery were 141.87 ± 34.48, 100.87 ± 26.48, and 37.49 ± 56.39, respectively, indicating a significant improvement in the patients’ symptoms after surgery (*P* < 0.001). The total mean score of ICIQ-FLUTSsex was 3.67 ± 3.63 (range, 0–10). In total, 22 (28.2%) women had an ICIQ-FLUTSsex score of 0, indicating no problems. The attachment of the rectovaginal fascia to the pericervical rings can be an effective surgical technique for correcting posterior vaginal wall prolapses, without significant morbidity. The PFDI-20 score improved significantly from before surgery to 12 months and 5 years after surgery.

## INTRODUCTION

Pelvic organ prolapse (POP) is one of the most common gynecological disorders that can severely affect the physical, social, and sexual activity of women [1]. POP can be diagnosed based on the symptoms or during physical examination, as an abnormal bulge of the vaginal wall beyond the hymen. Most women experience POP symptoms when the leading edge of the bulge reaches 0.5 cm distal to the hymenal ring [2]. The development of POP is influenced by genetic, anatomical, lifestyle, physiological, and reproductive factors [3]. Women with POP often have dysfunction in multiple compartments of the pelvic floor, such as the anterior, apical, and posterior vaginal walls [4]. However, many women with POP are asymptomatic, and the prevalence of POP based on symptoms is much lower (about 3–6%) than that based on examination (about 41–50%) [5].

The use of local or systemic estrogen for the prevention or treatment of POP is limited. However, some clinicians suggest that local estrogen can reduce the vaginal irritation associated with POP [6]. Moreover, vaginal pessaries can be successfully fitted for 92% of women with POP who want to preserve their fertility. Ring pessaries are more effective for treating stage II and III prolapses, while Gellhorn pessaries are more commonly used for stage IV prolapse. In 2–9% of patients, the pessaries may cause local devascularization or vaginal mesh erosion due to pressure [7].

Although anatomical correction is widely practiced, there is limited data on its success and the improvement of sexual symptoms. Therefore, it is essential to analyze and evaluate the patients’ sexual function and quality of life after surgery [8]. Based on these issues, the aim of this study was to assess the quality of life, pelvic discomfort, and sexual function of patients who underwent posterior pericervical repair or level I–III surgical procedures for POP after 5 years of follow-up.

## MATERIAL AND METHODS

### Study population

This retrospective cohort study involved women with rectocele and/or enterocele defects who underwent surgery at the Imam Khomeini Hospital, Tehran, Iran, from 2014 to 2021. We included women who had symptoms of pelvic floor disorders and POP-Q (Pelvic Organ Prolapse Quantification) tests that showed enterocele and/or rectocele with or without anterior and/or apical vaginal prolapse. These patients either preferred surgical intervention or did not respond to conservative treatments. The exclusion criteria were obesity, cancer of any genital or abdominal organs, multiple sclerosis, and pelvic radiation therapy. Moreover, patients who failed to follow up were excluded from the analysis.

To achieve the objectives of the study, we used the Mann–Whitney test with 90% detection power for a normal distribution. We set non-inferiority equal to 3, a significance level of 0.05, and a s.d. of 1.5 for the studied population. The value of d was set to 0.05 based on previous studies [9]. Consequently, the sample size was determined to be 50 patients.

### Study protocol

In the first stage, the patients’ demographic information, medical history, and history of pelvic floor disorders, such as urinary, gastrointestinal, and genital problems, were recorded in a form. The symptoms of POP were assessed based on the definitions of the International Continence Society (ICS) and the International Urogynecological Association (IUGA) [10]. Posterior level 2 defects were treated by posterior colporrhaphy, in which the rectovaginal fascia was repaired, and the perineal body or level III DeLancey was attached to the uterosacral ligament or level I DeLancey [10].

We used the Iranian version of the Pelvic Floor Distress Inventory-20 (PFDI-20) to measure the quality of life of patients with pelvic floor disorders due to their symptoms. The PFDI-20 is a valid questionnaire that evaluates the quality of life and pelvic discomfort in terms of bowel, bladder, or pelvic symptoms. Each subscale has six to eight questions about the patient’s symptoms, which are scored from 0 (no problem) to 4 (very bothersome). The scores of each subscale are summed, multiplied by 25, and divided by the number of questions. Each subscale ranges from 0 to 100, and the total PFDI-20 score ranges from 0 to 300. A higher score indicates a lower quality of life. The reliability and validity of this questionnaire in Iran have been confirmed by Hakimi *et al*. [11]. The patients completed this questionnaire before, 12 months, and 5 years after the surgery.

The International Consultation on Incontinence Questionnaire Female Sexual Matters Associated with Lower Urinary Tract Symptoms Module (ICIQ-FLUTSsex) is a questionnaire that assesses the impact of lower urinary tract symptoms on female sexuality and quality of life. It is widely used in research and clinical practice around the world. The ICIQ-FLUTSsex is derived from the Bristol Female Lower Urinary Tract Symptoms (BFLUTS) questionnaire, which is fully validated. The ICIQ-FLUTSsex consists of four items: 1) vaginal dryness causing pain or discomfort; 2) impact of urinary symptoms; 3) pain during sexual activity; and 4) urine leakage during sexual activity. The scores range from 0 to 14, with higher values indicating more severe symptoms. The questionnaire also includes bother scales for each item, which are not part of the total score but indicate the impact of individual symptoms [12]. The reliability and validity of this questionnaire in Iran have been confirmed by Pourmomeny *et al*. [13]. The patients completed this questionnaire 5 years after the surgery.

The study population completed the PFDI-20 questionnaire after the surgery. At the 5-year follow-up, the patients also completed the ICIQ-FLUTSsex questionnaire in addition to the PFDI-20 questionnaire.

### Statistical analysis

Descriptive data were presented as means, standard deviations, and/or percentages. The normality of the data was tested by the Shapiro–Wilk test before analysis. Continuous quantitative variables with normal distributions were compared using Student’s *t*-test and the Wilcoxon test. The analyses were performed using SPSS software (version 22, Chicago, IL, USA). A *P* value of less than 0.05 was considered statistically significant.

## RESULTS

We enrolled 460 patients who were eligible for vaginal surgery due to pelvic floor disorders. Of them, 180 patients who had posterior vaginal wall prolapse with or without other compartment prolapses underwent vaginal native tissue surgery. Only 107 patients were followed up for at least 12 months. The subjects’ ages ranged from 24 to 68 years, with a median of 50 years (49.69 ± 10.95). The parity of the patients was as follows: one (*n* = 3; 2.8%), two (*n* = 22; 20.56%), three (*n* = 17; 15.88%), four (*n* = 25; 23.36%), more than four (*n* = 40; 37.38%). In total, 52 patients (48.59%) were menopausal and one patient (0.93%) had a previous abdominal hysterectomy. The mode of delivery was vaginal (*n* = 96; 89.71%) or vaginal and cesarean section (*n* = 11; 10.28%).

Of the 107 patients, only 78 patients with a mean age of 56.16 ± 9.94 years (range, 29–73 years) were followed up for 5 years. Five patients had died, and 24 patients could not be contacted by phone. All patients had surgical repair of posterior vaginal compartments (rectoceles and/or enteroceles), either alone or along with surgery on other vaginal compartments. The average PFDI-20 scores before, 12 months, and 5 years after surgery were 141.87 ± 34.48, 100.87 ± 26.48, and 37.49 ± 56.39, respectively, showing a significant improvement in the patients’ symptoms after surgery (*P* < 0.001). The scores of each subscale of the PFDI-20 are presented in Table 1.

**Table 1:** Preoperative and postoperative PFDI-20 scores

Variable	Preoperative (*n* = 107)	After 12 months (*n* = 107)	After 5 years (*n* = 78)	*P* value
Pelvic Organ Prolapse Distress Inventory 6 (POPDI-6) (mean ± s.d.)	36.51 ± 13.70	20.54 ± 98.22	12.92 ± 24.06	<0.001
Colorectal-Anal Distress Inventory 8 (CRAD-8) (mean ± s.d.)	38.65 ± 12.69	22.83 ± 16.50	8.17 ± 16.74	<0.001
Urinary Distress Inventory 6 (UDI-6) (mean ± s.d.)	66.71 ± 24.09	57.50 ± 16.49	16.39 ± 21.81	<0.001
Total (mean ± s.d.)	141.87 ± 34.48	100.87 ± 26.48	37.49 ± 56.39	<0.001

Out of the 78 patients who were followed up for 5 years, only two (2.6%) reported vaginal noise, and 76 (97.4%) had no problem with this issue. Moreover, three patients (3.8%) had complications from the surgery and underwent reoperation within 2 months, whereas 75 patients (96.2%) did not need reoperation. The results of the ICIQ-FLUTSsex are shown in Table 2. The mean score of the ICIQ-FLUTSsex was 4.74 ± 3.63 (range, 0–14) before surgery and 3.67 ± 3.63 (range, 0–10) after surgery. The ICIQ-FLUTSsex scores before and 5 years after surgery indicated a significant improvement in the patients’ symptoms after surgery (*P* < 0.001). Among the items of the ICIQ-FLUTSsex, only the item of vaginal dryness causing pain or discomfort showed a significant improvement in the patients’ symptoms after surgery (*P* = 0.05). Furthermore, 20 patients (25.64%) did not have sexual intercourse. In total, 22 women (28.2%) had a score of 0 on the ICIQ-FLUTSsex, meaning they had no problems.

**Table 2:** ICIQ-FLUTSsex scores

Variable	Preoperative (*n* = 107)			After 5 years (*n* = 78)			*P* value
	Score (mean ± s.d.)	Minimum	Maximum	Score (mean ± s.d.)	Minimum	Maximum	
Pain or discomfort caused by dry vagina	0.76 ± 0.99	0	3	1.37 ± 1.58	0	4	0.05
Impact of urinary symptoms	0.29 ± 0.70	0	3	0.25 ± 0.91	0	4	0.133
Pain during sexual activity	1.56 ± 1.66	0	4	1.68 ± 1.73	0	4	0.607
Urine leakage during sexual activity	1.15 ± 1.73	0	4	1.65 ± 1.90	0	5	0.356
Total	3.67 ± 3.63	0	10	4.74 ± 3.64	0	14	<0.001

All patients who underwent surgery had posterior compartment prolapse of grade 2 or higher at the preoperative examination, and the prolapse improved in all cases after the surgery (*P* > 0.001). According to the results, seven of these patients did not have a history of stress urinary incontinence (SUI). However, after prolapse reduction, they had a positive cough test and a urodynamic study that showed SUI. Therefore, they underwent SUI surgery simultaneously with the prolapse surgery. All seven patients had complete resolution of SUI after the surgery. The combination of SUI surgery and surgery of other vaginal compartments at the same time did not show significant differences in preoperative and postoperative symptoms (the preoperative and postoperative scores for the SUI question on the PFDI-20 questionnaire were 3.42 ± 1.77 and 1.24 ± 0.82, respectively; *P* < 0.001). On the PFDI-20 questionnaire, three patients with SUI reported moderate urinary symptoms before the surgery, but these symptoms persisted after the surgery.

## DISCUSSION

There is a lack of data on the outcomes of POP surgery and the changes in sexual symptoms over time. It is important to analyze and evaluate the sexual disorders and quality of life of patients after the surgery over a long period. Therefore, this study assessed the quality of life, pelvic discomfort, and sexual function of patients 5 years after posterior pericervical repair. The results of the study showed that posterior compartment surgery improved the overall PFDI-20 score significantly from before the surgery to 12 months and 5 years after the surgery, compared to each of the other vaginal compartment surgeries individually. Moreover, the patients’ sexual function based on ICIQ-FLUTSsex improved significantly 5 years after the surgery compared to before the surgery.

The Food and Drug Administration has not approved any surgical mesh products for vaginal repair of POP owing to concerns about the side effects of using mesh in this area [14]. Therefore, surgeons have tried to describe a novel surgical technique for repairing rectoceles without mesh in one of the largest case series available [15]. This technique essentially combines the transvaginal and transanal approaches from gynecology and surgery, respectively. Compared to the pooled analyses of either transvaginal or transanal approaches, this technique provides more significant anatomical success and symptomatic relief in the medium term [16]. A review study suggested that uterosacral ligaments can offer considerable support for women with high rectoceles or rectoceles accompanied by posterior enteroceles, as they attach the posterior vaginal wall in full thickness (DeLancey level 3) [17].

Sohbati *et al*. reported that attaching the rectovaginal fascia to the pericervical ring in posterior vaginal wall prolapse repair was an effective surgical intervention with minimal morbidity, based on their short-term follow-up [10]. They also found that the surgery improved symptoms such as bulging, obstructed defecation, splinting, and incomplete bowel emptying significantly after 12 months. Moreover, the patients’ scores on the PFDI-20 questionnaire and clinical examinations improved significantly after 12 months [10]. However, the long-term outcomes of this operation are very important to determine. Therefore, this study examined the results of surgery after 5 years of follow-up. The results showed that the PFDI-20 score improved significantly 5 years after the surgery.

To assess subjective outcomes, we used validated questionnaires to measure symptoms related to pelvic floor disorders. Dietz *et al*. [18] and Ghanbari *et al*. [19] evaluated the functional outcome of sacrospinous hysteropexy after a mean follow-up of 12.7 and 18.62 months, respectively. They reported improvements in all domains of quality of life and pelvic floor function, except for pain and fecal incontinence. In another study, the effect of POP surgery on symptoms of obstructed defecation was evaluated at a 12-week follow-up. The PFDI-20 and POP-Q results showed significant improvements in splinting, straining, and incomplete emptying during defecation [20]. Our results were consistent with their findings, as we found significant improvements in PFDI-20 scores after 5 years. Henn *et al*. examined the outcomes of plication of the anterior rectal wall during posterior vaginal compartment repair, using a transvaginal technique and suturing the rectal muscularis layer in a zig-zag pattern caudally. They found significant improvements in symptoms of posterior vaginal compartment prolapse, such as bulging, obstructed defecation, and PFDI-20 score, after 27 months of follow-up [15]. Bastani *et al*. compared anterior and posterior sacrospinous ligament fixation in patients with apical compartment POP. Both groups showed a significant decrease in the postoperative UDI-6 and PFDI-20 compared to the preoperative status, after a follow-up of 6–18 months [21]. As reported in the literature, patients rarely receive long-term follow-up after prolapse surgery. The results of this study showed that the PFDI-20 questionnaire scores and the overall score of the patients improved in three domains after 5 years.

In this study, most participants had vaginal delivery rather than both vaginal childbirth and cesarean section. A study involving 5,236 Swedish women showed that vaginal delivery increased the risk of urinary incontinence by 275% for 10 years and 67% for 20 years after childbirth, compared to cesarean section [22]. Several factors can influence the onset of urinary incontinence, including the type of childbirth and pregnancy itself. Another study involving 220 Brazilian women found that cesarean sections did not prevent urinary incontinence for 2 years [23]. Urinary incontinence has a serious impact on the quality of life of many women, according to the ICIQ-SF. A qualitative study suggested that urinary incontinence changed women’s daily behavior, imposed restrictions, and even compromised their social lives [24]. Women with incontinence were also affected in their sexual activity. Intercourse can make many women feel embarrassed due to the possibility of experiencing urine loss. Some women may experience pain or discomfort in the vaginal area, besides their worries about urine loss [25]. Based on the study by Andrade *et al*., the ICIQ-FLUTSex is a valid and easy-to-use tool for assessing sexual dysfunction in women who complain of urinary incontinence [26]. The results of the ICIQ-FLUTSex in this study showed that the total mean score of the ICIQ-FLUTSex improved after 5 years. Therefore, the pericervical repair of level I–III surgical procedures can improve patients’ sexual function and quality of life.

Prolapse and obstructive defecation disorders can be treated with various surgical techniques that correct the anatomical defects and improve the obstructive symptoms. However, POP surgery has a high lifetime risk (11.1%) and a high rate of reoperations in women [27]. Rectocele repair can be performed by posterior colporrhaphy, site-specific defect repair, or sacrocolpoperineopexy. Posterior colporrhaphy is a traditional gynecological technique that has been used for more than 100 years [28]. However, transanal repairs are associated with fecal incontinence according to a review of different surgical methods [29]. Both transanal and transvaginal repairs improve prolapse symptoms and defecatory disorders, but transvaginal repair has better anatomical outcomes. Sacrocolpoperineopexy repair may worsen the symptoms of defecation disorders, according to some studies [11]. This study used the repair of the rectovaginal fascia and its connection to the cervical ring to treat rectoceles in women. This is the first time that the outcomes of this surgical technique have been evaluated for 5 years, as the researchers had limited resources.

A limitation of this study is the small sample size, which limits the generalizability of the results. Moreover, the ICIQ-FLUTSex was only administered to the patients after 5 years. Future studies with larger sample sizes and ICIQ-FLUTSex follow-up before and after surgery are needed to provide additional epidemiological information and to develop more appropriate and comprehensive solutions.

## CONCLUSION

This study showed that attaching the rectovaginal fascia to the pericervical rings was an effective surgery for correcting posterior vaginal wall prolapses, with minimal morbidity. The surgery improved the PFDI-20 score significantly from before the surgery to 12 months and 5 years after the surgery. There is a need for clinical trials with long-term follow-up to compare the efficacy of this technique with other surgical procedures for repairing posterior vaginal wall prolapses. Moreover, our team has examined the manometric results of patients who underwent posterior pericervical repair or level I–III surgery and will present the findings in a future paper.

## References

[1] Raju R, Linder BJ (2021). Evaluation and management of pelvic organ prolapse. Mayo Clinic Proceedings.

[2] American College of Obstetricians and Gynecologists and the American Urogynecologic Society (2019). INTERIM UPDATE: This Practice Bulletin is updated as highlighted to reflect the US Food and Drug Administration order to stop the sale of transvaginal synthetic mesh products for the repair of pelvic organ prolapse. Pelvic Organ Prolapse. Female Pelvic Med Reconstr Surg.

[3] Vergeldt TF, Weemhoff M, IntHout J, Kluivers KB (2015). Risk factors for pelvic organ prolapse and its recurrence: a systematic review. Int Urogynecol J.

[4] Cattani L, Decoene J, Page A-S, Weeg N, Deprest J, Dietz HP (2021). Pregnancy, labour and delivery as risk factors for pelvic organ prolapse: a systematic review. Int Urogynecol J.

[5] Weintraub AY, Glinter H, Marcus-Braun N (2019). Narrative review of the epidemiology, diagnosis and pathophysiology of pelvic organ prolapse. International Braz J Urol.

[6] Radnia N, Hajhashemi M, Eftekhar T, Deldar M, Mohajeri T, Sohbati S (2019). Patient satisfaction and symptoms improvement in women using a vginal pessary for the treatment of pelvic organ prolapse. J Med Life.

[7] Tam M-S, Lee VY, Ellen L, Wan RS, Tang JS, He JM (2019). The effect of time interval of vaginal ring pessary replacement for pelvic organ prolapse on complications and patient satisfaction: A randomised controlled trial. Maturitas.

[8] Van den Broeck S, Nullens S, Jacquemyn Y, De Schepper H, Vermandel A, Komen N (2023). Posterior compartment prolapse and perineal descent: systematic review of available support devices. Int Urogynecol J.

[9] Haylen BT, Maher CF, Barber MD, Camargo S, Dandolu V, Digesu A (2016). An International Urogynecological Association (IUGA)/International Continence Society (ICS) joint report on the terminology for female pelvic organ prolapse (POP). Int Urogynecol J.

[10] Sohbati S, Hajhashemi M, Eftekhar T, Deldar M, Radnia N, Ghanbari Z (2020). Outcomes of Surgery with Vaginal Native Tissue for Posterior Vaginal Wall Prolapse Using a Special Technique. J Med Life.

[11] Hakimi S, Hajebrahimi S, Bastani P, Aminian E, Ghana S, Mohammadi M (2023). Translation and validation of the pelvic floor distress inventory short form (PFDI-20), Iranian version. BMJ Womens Health.

[12] Przydacz M, Dudek P, Chlosta P (2021). Polish versions of the ICIQ-FLUTS and the ICIQ-FLUTS LF: translation, adaptation, and validation of female-specific instruments for evaluation of lower urinary tract symptoms. Int Urogynecol J.

[13] Pourmomeny A, Alebouye-Langeroudi S, Zargham M (2018). Reliability and validity of the Persian Language version of the female lower urinary tract symptoms' long form questionnaire. Iranian Journal of Nursing and Midwifery Research.

[14] Farmer Z-L, Domínguez-Robles J, Mancinelli C, Larrañeta E, Lamprou DA (2020). Urogynecological surgical mesh implants: New trends in materials, manufacturing and therapeutic approaches. Int J Pharm.

[15] Henn EW, Cronje HS (2018). Rectocele plication: description of a novel surgical technique and review of clinical results. Int Urogynecol J.

[16] Kulik V, Kriv A, Aleni M (2020). How to Optimize Surgical Treatment of Chronic Anal Fissure Combined With Rectocele In Women. Systematic Reviews in Pharmacy.

[17] Karram M, Maher C (2013). Surgery for posterior vaginal wall prolapse. Int Urogynecol J.

[18] Dietz V, Huisman M, de Jong JM, Heintz PM, van der Vaart CH (2008). Functional outcome after sacrospinous hysteropexy for uterine descensus. Int Urogynecol J.

[19] Ghanbari Z, Veisi F, Eftekhar T, Deldar M, Mostaan F, Adabi K (2023). Concomitant pericervical reconstruction with sacrospinous hysteropexy: Anatomical and functional results. Taiwan J Obstet Gynecol.

[20] Grimes CL, Overholser RH, Xu R, Tan-Kim J, Nager CW, Dyer KY (2016). Measuring the impact of a posterior compartment procedure on symptoms of obstructed defecation and posterior vaginal compartment anatomy. Int Urogynecol J.

[21] Bastani P, Ebrahimpour M, Mallah F, Hajebrahimi S, Salehi Pourmehr H (2021). Comparison of the Functional and Anatomical Outcomes of Abdominal Sacrocolpopexy and Vaginal Sacrospinous Ligament Suspension for the Treatment of Apical Prolapse. Journal of Obstetrics Gynecology and Cancer Research.

[22] Gyhagen M, Bullarbo M, Nielsen TF, Milsom I (2013). The prevalence of urinary incontinence 20 years after childbirth: a national cohort study in singleton primiparae after vaginal or caesarean delivery. BJOG.

[23] De Carvalho KB, Ibiapina FTO, Machado DdCD (2021). Força muscular do assoalho pélvico em mulheres com queixas de disfunção pélvica. Fisioterapia Brasil.

[24] Henkes DF, Fiori A, Carvalho JAM, Tavares KO, Frare JC (2015). Incontinência urinária: o impacto na vida de mulheres acometidas e o significado do tratamento fisioterapêutico [Urinary incontinence: the impact on the lives of affected women and the significance of physiotherapy treatment]. Semina: Ciências Biológicas e da Saúde.

[25] de Caldas Reis LE, Costa MM, Iocca DC, Siqueira SdCF, de Medeiros Queiroz J, Sampaio JMC (2023). Incontinência urinária em mulheres idosas e qualidade de vida: sua relação com o fortalecimento do assoalho pélvico. Brazilian Journal of Development.

[26] Andrade BFd, Katz L, Rangel AEdO, Guendler JdA (2020). Assessment on the measurement properties of the Brazilian Portuguese language version of the International Consultation on Incontinence Questionnaire Female Sexual Matters Associated with Lower Urinary Tract Symptoms Module (ICIQ-FLUTSsex). Revista Brasileira de Saúde Materno Infantil.

[27] Kayondo M, Geissbüehler V, Migisha R, Kajabwangu R, Njagi J, Kato PK (2022). Risk factors for recurrence of pelvic organ prolapse after vaginal surgery among Ugandan women: a prospective cohort study. Int Urogynecol J.

[28] Guzman-Negron JM, Fascelli M, Vasavada SP (2019). Posterior vaginal wall prolapse: suture-based repair. Urologic Clinics.

[29] Grimes CL, Schimpf MO, Wieslander CK, Sleemi A, Doyle P, Wu Y (2019). Surgical interventions for posterior compartment prolapse and obstructed defecation symptoms: a systematic review with clinical practice recommendations. Int Urogynecol J.

